# IL-22-producing CD4+T cells in the treatment response of rheumatoid arthritis to combination therapy with methotrexate and leflunomide

**DOI:** 10.1038/srep41143

**Published:** 2017-01-24

**Authors:** Wei Zhong, Ling Zhao, Tao Liu, Zhenyu Jiang

**Affiliations:** 1 The First Hospital of Jilin University, Department of Rheumatology, Changchun, 130021, China

## Abstract

T cells are key players in immune-mediated rheumatoid arthritis (RA). We previously reported that interleukin (IL)-22^+^CD4^+^T helper (IL-22^+^ Th) cells and IL-22 critically control the pathogenesis of RA. Here we monitored circulating levels of different IL-22^+^ Th cell subsets and measured plasma levels of IL-22, IL-17, and interferon (IFN)-γ in 60 patients with active RA following 12-week combination methotrexate (MTX) and leflunomide (LEF) therapy (MTX+LEF) and 20 healthy individuals. We found the frequencies of circulating IFN-γ^−^IL-17^−^IL-22^+^ (Th22), IFN-γ^−^IL-17^+^ (total Th17), IFN-γ^+^IL-17^−^IL-22^+^ (IL-22^+^Th1) cells, and IFN-γ^−^IL-17^+^IL-22^+^ (IL-22^+^Th17) cells, as well as the plasma levels of IL-22, IL-17 and IFN-γ to be significantly reduced in RA patients that responded to treatment, but not in non-responders. Reductions in plasma IL-22 level significantly correlated with percentage of circulating Th22 cells and the decrease of plasma IL-22 level correlated with the reduction of DAS28 in responders. Our data suggests that circulating Th22 cells and plasma IL-22 level play a detrimental role in RA. The combination MTX+LEF therapy, by targeting Th22 cells and reducing IL-22 level, relieves the immune defects and ameliorates symptoms of RA. This study provides novel mechanistic understanding of the pathogenesis of RA, which may promote a design of better therapies for RA.

Rheumatoid arthritis (RA) is a common inflammatory disorder manifested as progressive joint destruction, dysfunction, deformity, and eventually disability. At the cellular level, RA is characterized by infiltration of a variety of immune cells into the synovial membrane, where the crosstalk among distinct immune cell subsets, cytokines secreted by these cells, and synovial fibroblasts leads to sustained inflammation, autoimmune responses, and subsequent damage to bones and cartilage^1^. Understanding the immune dysfunction of RA may aid rational design of treatments targeting the disease.

The cytokine interleukin (IL)-22 is a member of the IL-10 family. By activating proliferative pathways and inhibiting apoptotic pathways, IL-22 significantly controls tissue responses to inflammation^2^. Several types of immune cells, most notably, three subsets of CD4^+^T helper (Th) cells: IFN-γ^−^IL-17^−^IL-22^+^ (Th22), IFN-γ^−^IL-17^+^IL-22^+^ (IL-22^+^Th17), and IFN-γ^+^IL-17^−^IL-22^+^ (IL-22^+^Th1) cells are responsible for IL-22 production^3^,^4^. In humans, Th22 cells are the major Th subset responsible for IL-22 production in the peripheral circulation, accounting for approximately 37–63% of circulating IL-22^+^cells^4^. Th22 cells express neither IL-17 nor INF-γ and thus can be identified by flow cytometry as IFN-γ^−^IL-17^−^IL-22^+^cells. Th17 cells, are a IL-17A-positive but IFN-γ- negative pro-inflammatory CD4^+^Th subset demonstrated to contribute to RA pathology^5^. Th1 cells are the major source of pro-inflammatory cytokines, such as IFN-γ and tumor necrosis factor (TNF)-α. However, the significance of these cells in the development of autoimmune diseases, such as RA or systemic lupus erythematosus (SLE), remains controversial^6^,^7^,^8^,^9^,^10^. Recently, a few studies suggest the pro-inflammatory/pathogenic role of IL-22 in the onset and development of RA. In the animal model mimicking RA in human, IL-22 plays an important role in the productions of inflammatory components, hampering Th1 plasticity and favoring Th17 maintenance and survival, pointing to the potential therapeutic benefits by blocking IL-22 in preventing immune-complex deposition and joint destruction in RA patients^11^,^12^. In addition, IL-22 significantly enhances the proliferation and activation of fibroblast-like synoviocytes, suggesting its contribution to the synovium hyperplasia during RA progression^13^,^14^. We previously reported that the percentages of circulating Th22, IL-22^+^Th1, and IL-22^+^Th17 cells, and serum IL-22 levels were significantly higher in RA patients than in healthy individuals^15^, suggesting that the major IL-22-producting CD4^+^Th cells may act through the overproduction of IL-22 to stimulate the pathogenesis of RA.

The major medication for RA is the disease-modifying antirheumatic drugs (DMARDs), including methotrexate (MTX), leflunomide (LEF), sulfasalazine, and hydroxychloroquine. MTX is the most commonly used DMARD for RA, and is often administered in combination with other DMARDs^16^. MTX was previously reported to induce apoptosis of activated CD4^+^T cells^17^,^18^, inhibit Th cell signaling in psoriasis, and downregulate Th-related mRNA expression^19^. LEF is reported to inhibit pyrimidine biosynthesis, to suppress B cell antibody responses^20^, and to shift the Th1/Th2 balance from a preferential pro-inflammatory Th1 response to an immune-modulatory Th2 response^21^. Although it can be used alone, LEF is also used in combination with MTX for patients not responding to MTX treatment alone.

To further characterize the significance of IL-22 and IL22^+^CD4^+^T cells in RA, particularly in the treatment response of RA patients to DMARDs, we carried out a prospective study and monitored the levels of circulating IL-22-producing Th cells and the plasma IL-22 level in RA patients following treatment with combination MTX+LEF therapy.

## Result

### Treatment significantly lessened disease activity and alleviated RA-specific clinical indicators in some but not all RA patients

In this study, we recruited 60 newly diagnosed RA patients and 20 age- and gender-matched healthy individuals (HC). Upon recruitment and before administration of any treatment, all RA patients presented moderate to high disease activity, as demonstrated by a mean DAS28 value of 5.98 (range: 3.29–9.33). Consistently, levels of CCP, CRP, ESR, and RF were also significantly higher in these patients than healthy controls (P < 0.05; Table 1).

**Figure 1:**
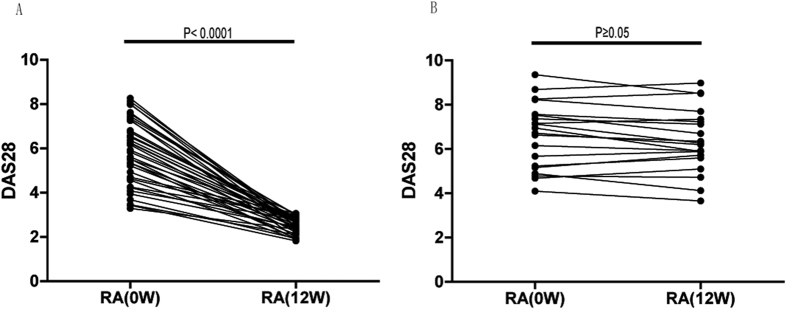
DAS28 scores were significantly improved in RA responders (n = 40) but not in RA non-responders (n = 20) after the combination methotrexate (MTX)+ leflunomide (LEF) therapy. DAS28 values were compared between pre- and post-treatment in each RA responder (**A**) or non-responder (**B**).

In response to combination MTX+LEF treatment (Table 2), the DAS28 of 40 RA patients fell from 5.72 (range: 3.29–8.26) before treatment to 2.60 (range: 1.82–3.05) after treatment (P < 0.0001, Fig. 1A) (response group), while a treatment response was not detected in the remaining 20 patients (non-response group), in which the post-treatment DAS28 of 6.23 (range: 3.65–8.98) did not differ significantly from that before treatment (DAS28 = 6.92 (range: 4.10–9.33); P ≥ 0.05, Fig. 1B). Consistently, RA-specific clinical indicators, including RF, CCP, ESR, and CRP were significantly reduced after treatment only in the response group, as compared to the values before the treatment (P < 0.05) or to those in the non-response group after the treatment (P < 0.05). Specifically, ESR and CRP were reduced in the response group to the levels comparable to those in HC (P ≥ 0.05). At the baseline level, that is, before treatment, these factors did not differ dramatically between the response group and non-response group (P ≥ 0.05). Furthermore, the levels of these clinical indicators were not significantly changed in the non-response group (P ≥ 0.05; Table 2).

### Treatment significantly reduced circulating Th22 cells, total Th17 cells, and IL-22^+^Th17 cells, but not IL-22^+^Th1 cells in RA patients responsive to treatment

**Figure 2:**
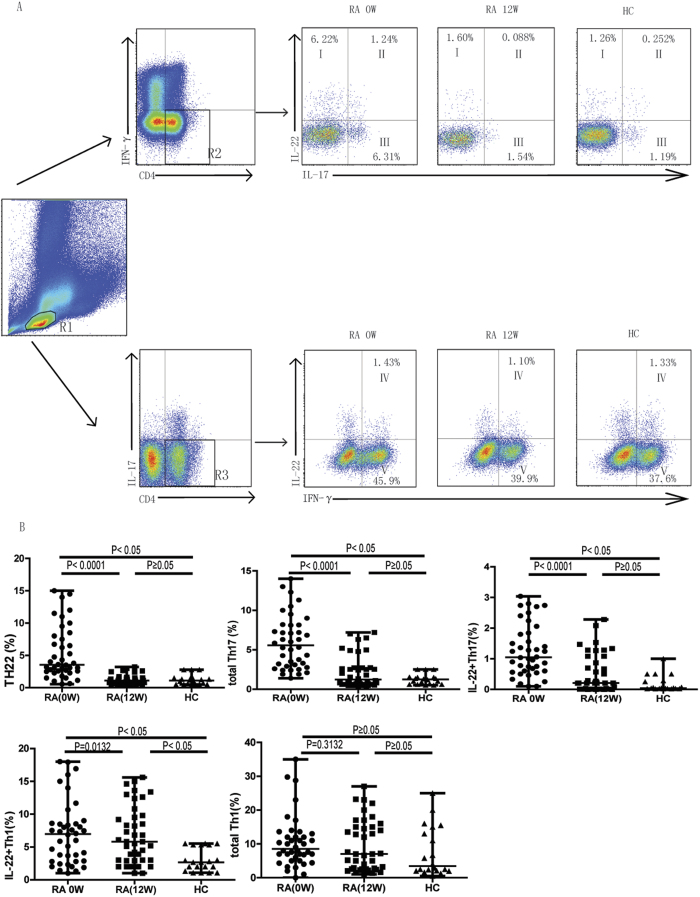
The percentages of circulating Th22, total Th17, and IL-22^+^Th17, IL22^+^Th1, but not total Th1 cells decreased following MTX+LEF therapy only in patients that exhibited clinical improvement (n = 40). Peripheral blood mononuclear cells (PBMCs) were collected from RA patients responsive to treatment at baseline (before treatment, 0 week) and after treatment (12 weeks) with MTX+LEF, and analyzed by flow cytometry for the percentage of different Th subsets, including IFN-γ^−^IL-17^−^IL-22^+^ (Th22), IFN-γ^−^IL-17^+^ (total Th17), IFN-γ^−^IL-17^+^IL-22^+^ (IL-22^+^Th17), IFN-γ^+^IL-17^−^ (total Th1), and IFN-γ^+^IL-17^−^IL-22^+^ (IL-22^+^Th1) cells. (**A**) The gating strategy for detecting different subsets of Th cells and representative flow images on samples from RA patients at 0 week (0 W), 12 week (12 W), or from healthy controls (HC; n = 20). R1, lymphoctyes; R2, IFN-γ^−^CD4^+^T cells; R3, IL-17^−^CD4^+^T cells; I, Th22 cells; II, IL-22^+^Th17 cells; III, IL-22^−^Th17 cells; IV; IL-22^+^Th1 cells; V; IL-22^−^Th1 cells.

**Figure 3:**
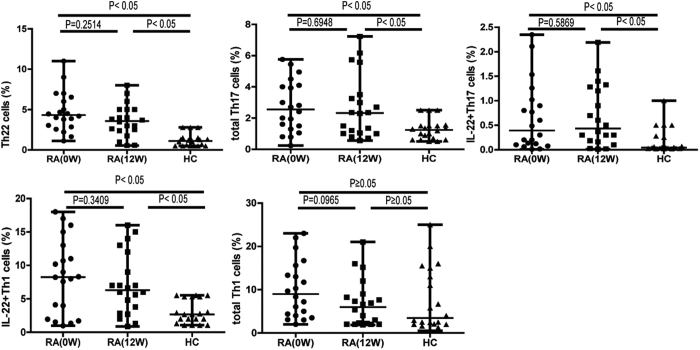
The percentages of circulating Th22, total Th17, IL-22^+^Th17, IL22^+^Th1, or total Th1 cells did not decrease following the combination MTX+ leflunomide LEF therapy in non-responding RA patients (n = 20). PBMCs were collected from RA patients resistant to treatment at baseline (before treatment, 0 week) and after treatment (12 weeks) with MTX+LEF, and analyzed by flow cytometry for the percentage of different Th subsets, including IFN-γ^−^IL-17^−^IL-22^+^ (Th22), IFN-γ^−^IL-17^+^ (total Th17), IFN-γ^−^IL-17^+^IL-22^+^ (IL-22^+^Th17), IFN-γ^+^IL-17^−^ (total Th1), and IFN-γ^+^IL-17^-^IL-22^+^ (IL-22^+^Th1) cells.

To assess the impact of the combination MTX+LEF therapy on IL-22^+^CD4^+^T cells, we used flow cytometry to analyze circulating T cell subsets before and after treatment. Figure 1A showed the gating strategy used to detect IFN-γ^−^IL-17^−^IL-22^+^ (Th22) cells (upper panels, quadrant I), IFN-γ^−^IL-17^+^ (total Th17) cells, IFN-γ^−^IL-17^+^IL-22^+^ (IL-22^+^Th17) cells, IFN-γ^+^IL-17^−^ (total Th1) cells and IFN-γ^+^IL-17^−^IL-22^+^ (IL-22^+^Th1) cells. In the response group, the percentages of circulating Th22 cells, total Th17 cells, and IL-22^+^Th17 cells were dramatically reduced after treatment (P < 0.0001; Fig. 2B). In addition, treatment reduced the percentage of these Th cell subsets to levels comparable to that in HC (P ≥ 0.05; Fig. 2B). In contrast, the circulating IL-22^+^Th1 cells respond to MTX+LEF treatment and decrease to a level lower than that before the treatment (P = 0.0132) but was significantly higher than in HC (P < 0.05; Fig. 2B). Total Th1 cell counts were not changed during RA development, or in response to treatment (P ≥ 0.05; Fig. 2B). In the non-response group, no significant changes in T cell subset distributions were observed (P ≥ 0·05; Fig. 3).

### Treatment significantly lowered plasma levels of IL-22, IL-17, and IFN-γ in RA patients showing treatment response

**Figure 4:**
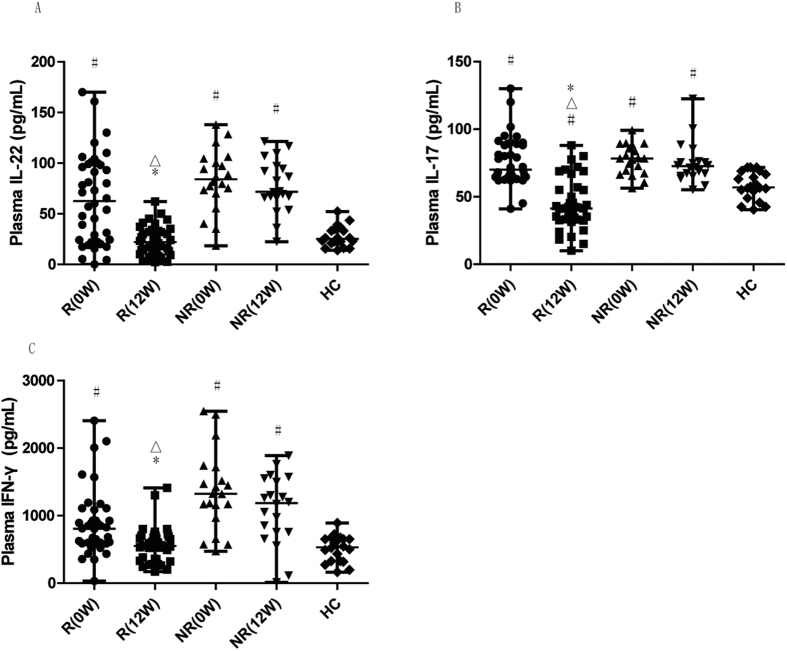
Plasma levels of IL- 22, IL-17 and IFN-γ decreased following MTX+LEF treatment in RA responders. The plasma levels of IL-22 (**A**), IL-17 (**B**), and IFN-γ (**C**) were measured by ELISA in RA responders and non-responders before treatment (0 W), after treatment (12 W), and in HC. R, response group; NR, non-response group. *P < 0.05 versus before treatment in response group. ^#^P < 0.05 versus healthy control. ^△^P < 0.05 versus non-response group at the same time point.

Since Th22, Th17, and Th1 cells are major sources of IL-22, IL-17, and IFN-γ, respectively, we further examined alterations in the levels of these cytokines in the treatment response of RA patients to MTX+LEF therapy. As shown in Fig. 4A to C, the plasma levels of all three cytokines were significantly reduced after treatment in the response group, as compared to the values before the treatment (P < 0.05). IL-22 and IFN-γ were reduced to levels comparable to those in HC (P ≥ 0.05 for IL-22 and IFN-γ), and IL-17 was reduced to a level significantly lower than that in HC (P < 0.05).

### Plasma IL-22 and IL-17 levels positively correlated with the percentage of circulating Th22 and Th17 cells, respectively, yet IFN-γ not correlated with the percentage of circulating Th1 cells in RA patients responsive to MTX+LEF treatment

**Figure 5:**
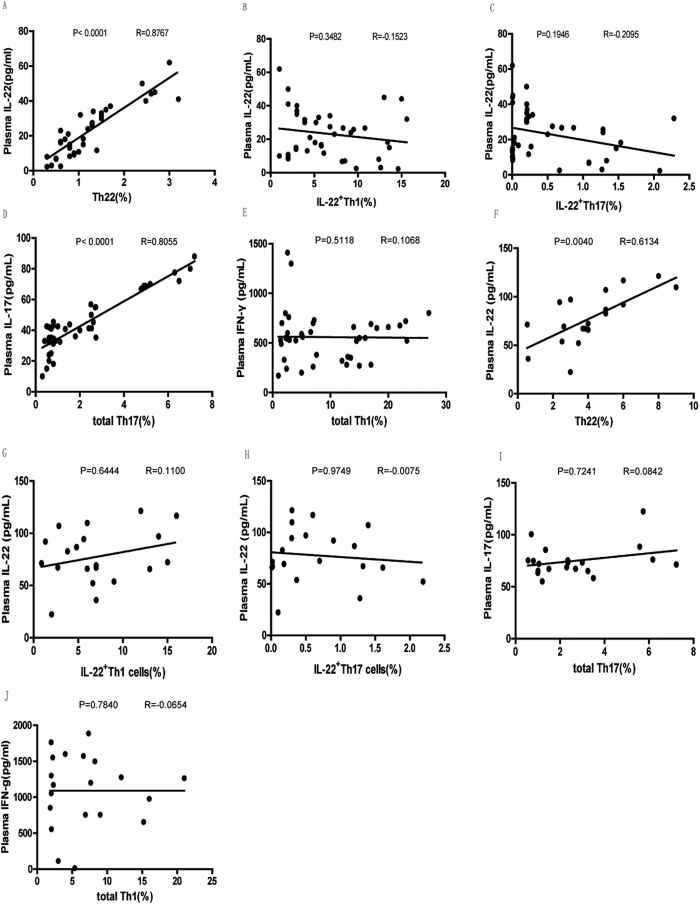
Plasma levels of IL-22 and IL-17 positively correlate with the percentage of circulating Th22 and total Th17 cells, respectively in RA responders while the plasma levels of IL-22 positively correlated with the percentage of circulating Th22 cells in RA non-responders. The correlation between the plasma IL-22 level and the percentage of circulating Th22 cells (**A**), the percentage of circulating IL-22^+^Th1 cells (**B**), or the percentage of circulating IL-22^+^Th17 cells (**C**) the correlation between the plasma level of IL-17 and the percentage of total circulatingTh17 cells (**D**), the correlation between the plasma level of IFN-γ and the percentage of total circulating Th1 cells (**E**) in RA responders; the correlation between the plasma IL-22 level and the percentage of circulating Th22 cells (**F**), the percentage of circulating IL-22^+^Th1 cells (**G**), or the percentage of circulating IL-22^+^Th17 cells (**H**) the correlation between the plasma level of IL-17 and the percentage of total circulatingTh17 cells (**I**); the correlation between the plasma level of IFN-γ and the percentage of total circulating Th1 cells (**J**) in RA non-responders after treatment were analyzed using Spearman’s rank correlation test.

To identify the source of serum IL-22, IL-17, and IFN-γ in RA patients responsive to MTX+LEF treatment, we performed correlation analysis (Fig. 5A–E). We found that plasma IL-22 levels positively and significantly correlated with the percentage of circulating Th22 cells (Fig. 5A; P < 0.0001, R = 0.8767), but not with that of IL22^+^Th1 (Fig. 5B; P = 0.3482, R = −0.1523) or IL22^+^Th17 cells (Fig. 5C; P = 0.1946, R = −0.2095) in RA patients responsive to treatment. Plasma IL-17 level positively and significantly correlated with the percentage of total circulating Th17 cells (Fig. 5D; P < 0.0001, R = 0.8055), while plasma IFN-γ level did not correlate with the percentage of total circulating Th1 cells (Fig. 5E; P = 0.5118, R = 0.1068). In RA patients non-responsive to MTX+LEF treatment (Fig. 5F–J), plasma IL-22 levels positively and significantly correlated with the percentage of circulating Th22 cells (Fig. 5F; P = 0.0040, R = 0.6134). No significant correlations were found between plasma IL-22 levels and IL22^+^Th1 (Fig. 5G; P = 0.6444, R = 0.1100) or IL22^+^Th17 cells (Fig. 5H; P = 0.9749, R = −0.0075), between plasma IL-17 level and total circulating Th17 cells (Fig. 5I; P = 0.7241, R = 0.0842), or between plasma IFN-γ level and total circulating Th1 cells (Fig. 5J; P = 0.7840, R = −0.0654) in non-response RA patient.

### DAS28 positively correlated with plasma IL-22, IL-17 levels and IL-22-producing Th cells in untreated RA, while the reduction of DAS28 in RA patients responsive to MTX+LEF treatment correlated with the decrease of the plasma level of IL-22

**Figure 6:**
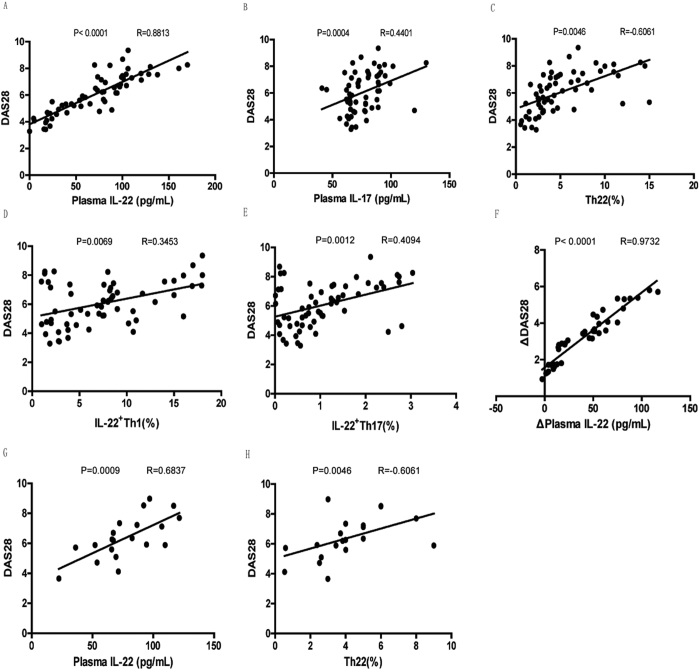
DAS28 positively correlated with plasma IL-22, IL-17 levels, and IL-22-producing Th cells in untreated RA, only with plasma IL-22 level in RA responders, and with plasma IL-22 level as well as the percentage of circulating Th22 cells in RA non-responders after treatment. The correlation between DAS28 and the plasma IL-22 level (**A**), the plasma level of IL-17 level (**B**), the percentage of circulating Th22 cells (**C**), the percentage of circulating IL-22^+^Th1 cells (**D**), or the percentage of circulating IL-22^+^Th17 cells (**E**) in untreated RA; the correlation between the decrease of DAS28 and the decrease of the plasma level of IL-22 (**F**) in RA responders after treatment; the correlation between the DAS28 and the plasma level of IL-22 (**G**), the percentage of circulating Th22 cells (**H**) in RA non-responders after treatment were analyzed using Spearman’s rank correlation test.

By analyzing the correlations between DAS28 and different parameters, we identified that DAS28 score positively correlated with plasma IL-22 (Fig. 6A; P < 0.0001, R = 0.8813), IL-17 (Fig. 6B; P = 0.0004, R = 0.4401) levels, and the frequencies of IL-22-producing Th cells, including Th22 (Fig. 6C; P = 0.0046, R = 0.6061), IL-22^+^Th1 (Fig. 6D; P = 0.0069, R = 0.3453), and IL-22^+^Th17 (Fig. 6E; P = 0.0012, R = 0.4094) in all RA patients before treatment. After treatment, no significant correlation between DAS28 value and plasma cytokines or the frequencies of circulating Th subsets was detected (P ≥ 0.05); however, a positive correlation between the reduction in DAS28 score and the decrease of plasma level of IL-22 was observed in RA patients responsive to treatment (Fig. 6F; P < 0.0001, R = 0.9732). In non-responding RA patients after treatment, DAS28 value positively correlated with plasma IL-22 level (Fig. 6G; P = 0.0009, R = 0.6837) and circulating Th22 cells (Fig. 6H; P = 0.0046, R = 0.6061), but no significant correlation was found between the change of DAS28 value and the change of cytokines (P ≥ 0.05) or Th subsets (P ≥ 0.05).

## Discussion

The role of IL-22 in inflammatory and autoimmune disorders remains controversial, with some studies suggesting a protective role^22^,^23^, while others, a pathogenic role^24^,^25^,^26^. We previously reported that the level of IL-22 and IL-22^+^CD4^+^T cells in the peripheral circulation were significantly higher in RA patients than in healthy individuals^15^, which may suggest a pathogenic role of these factors, but also could be attributed to a protective response of the body to RA development. In this study, to further characterize the significance of IL-22^+^CD4^+^T cells in the pathogenesis of RA, we treated RA patients with MTX plus LEF, and monitored circulating levels of IL-22^+^CD4^+^T subsets (including Th22, IL-22^+^Th17, and IL-22^+^Th1 cells) and plasma IL-22 in response to treatment. Our data showed that all three subsets and IL-22 levels were significantly higher in all RA patients than in HC and correlated with DAS positively before treatment. Following treatment, circulating Th22, total Th17, IL-22^+^Th17 cells and IL-22^+^Th1, as well as plasma levels of IFN-γ, IL-17, and IL-22 were significantly reduced only in patients responsive to treatment. In contrast, Th1 cell levels did not change following treatment in any RA patients. Furthermore, the decrease in plasma IL-22 levels significantly correlated with reduced levels of circulating Th22 cells. The decrease of plasma levels of IL-22 positively correlated with the decrease of DAS after treatment. Our data supports the theory that IL-22^+^CD4^+^T cells contribute to RA pathogenesis, and thus that targeting these cells may generate treatment responses among RA patients.

DMARDs are a group of drugs related only by their efficacy in targeting one or more processes underlying RA^27^. DMARDs include two major classes, synthetic and biological, and the former is further divided into conventional synthetic and targeted synthetic^28^. In contrast to the targeted synthetic DMARDs that target a specific molecular structure, the modes of action of conventional synthetic DMARDs remains largely unknown. MTX is a commonly used conventional synthetic DMARD, and the anchor drug in the management of RA^29^. The 2015 American College of Rheumatology Guideline for the Treatment of RA recommends using MTX to initiate treatment against RA^30^. Several randomized clinical trials have confirmed the efficacy of MTX as either a first-line or second-line DMARD to treat RA^31^,^32^,^33^. At the molecular and cellular level, MTX can down-regulate expression of chemokine CCL20 and cytokine IL22 in Th1, Th17 and Th22 cells, in patients with psoriasis^19^. Consistently, MTX significantly reduces serum IL-22 level in psoriasis^34^. LEF is an isoxazole derivative that critically controls the *de novo* synthesis of pyrimidine ribonucleotide uridine monophosphate^35^. LEF presents a potent therapeutic effect in a rat model of experimental autoimmune uveitis, which is associated with a decrease in serum levels of IL-17 and IFN-γ, as well as the number of Th17 cell^36^. Administration of both MTX and LEF to rats with type II collagen-induced arthritis reduced serum levels of IL-17, receptor activator of NF-kB ligand (RANKL), and osteoprotegerin (OPG)^37^. Large cohort studies indicate that combination therapy with MTX and LEF is efficacious and safe in the treatment of active RA^38^,^39^,^40^, and provides superior clinical benefits to MTX alone^39^. Therefore, we applied MTX and LEF combination therapy in this study.

To identify the immune mechanisms targeted by MTX+LEF combination therapy that may contribute to RA recovery, we monitored alterations in different circulating subsets of IL-22^+^CD4^+^Th cells and the plasma concentrations of cytokines IL-22, IL-17, and IFN-γ, in 60 patients with active RA, before and after a 12-week treatment course. We showed that 40 patients responded to treatment and achieved clinical amelioration, as represented by improvements in the disease activity indicator DAS28 and other clinical indicators including RF, CCP, ESR, and CRP, while the remaining 20 patients did not, reflecting the heterogeneous nature of the disease. Specifically, we found that the plasma IL-22 level in the response group decreased significantly following treatment, while in non-responders no significant changes in plasma IL-22 level was detected. In the response group the plasma IL-22 level was reduced to the baseline level detected in healthy individuals, and, notably, the decrease of plasma levels of IL-22 positively correlated with the reduction of DAS28. These results support the pathogenic role of elevated IL-22 in the development of RA, consistent with our previous findings^15^, suggesting that correcting/reducing IL-22 through MTX+LEF therapy can attenuate RA-related abnormalities. Since CD4^+^Th cells, including Th22 and IL-22-producing Th1 and Th17 cells^3^,^4^,^41^, are the major source of plasma IL-22, we measured circulating levels of these cells in responders and non-responders. The number of citrulline-specific Th1 cells has been reported to increase in the circulation of patients with RA^27^. Elevation of Th17 cells is also suggested to play an important role in the initiation and development of RA^42^. We found that Th22, IL-22^+^Th17, as well as IL-22^+^Th1 cells, exhibited the same pattern as plasma IL-22 in responders, but not in non-responders. Furthermore, a positive and significant correlation was identified between the plasma IL-22 level and the percentage of circulating Th22 cells in the drug-response patients. This data, when combined with our previous finding that in untreated RA patients, the frequencies of circulating IL-22^+^CD4^+^T cells and plasma IL-22 level are correlated, and are both significantly higher than in healthy individuals, indicates that IL-22 plays a significant role in the pathogenesis of RA.

In addition to plasma IL-22, we showed that the plasma level of IL-17 was significantly higher in RA patients than in healthy individuals and positive correlated with DAS28 before treatment, and was significantly reduced (to lower-than-baseline levels) following combination therapy. The alteration in IL-17 was significantly and positively correlated with changes in levels of IL-17-producing Th17 cells, indicating the significant role of Th17 cells in the pathogenesis of RA and in the response to MTX+LEF therapy, and consistent with previous findings^5^.

In a previous study, we found no significant difference in the levels of circulating Th1 cells between RA patients before treatment and healthy individuals. In this study, no obvious change was observed in these cells before and after treatment, yet dramatic reductions in plasma IFN-γ levels were noted only in responders. The implications of these data are multi-fold. First, Th1 cells may not be the major source of plasma IFN-γ, as supported by the non-significant correlation between the plasma level of IFN-γ and the percentage of total circulating Th1 cells we observed in this study. Other IFN-γ-producing cells, such as γδT cells, may be responsible for regulating plasma IFN-γ level following MTX+LEF treatment. Second, we identified significantly higher levels of circulating IL-22^+^Th1 cells in responders than in HC at the baseline level and these cells decreased after treatment in responsive RA patients, suggesting that up-regulated IL-22^+^Th1 cell levels may contribute the pathogenesis of RA and they may participate in response to MTX+LEF treatment. Third, we collected post-treatment samples immediately after 12 weeks of MTX+LEF. It may take longer for Th1 cells to respond to this treatment.

Although, in this study, 40 RA patients responded to MTX+LEF treatment, 20 failed to do so. More importantly, we observed no reductions in circulating Th22, Th17, IL-22^+^Th17 or plasma levels of IL-22, IL-17, or IFN-γ in non-responders, further supporting the significance of these cells and cytokines in determining response to MTX+LEF treatment in RA. Interestingly, in the non-responding group, we found that the level of plasma IL-22 correlated positively with the circulating Th22 cells, which suggested that Th22 cells may be the major source of plasma IL-22 in these patients. Furthermore, the plasma level of IL-22 and the frequency of Th22 cells correlated with DAS28 values in patients non-responsive to MTX+LEF after treatment. From these data, we inferred that Th22 and IL-22 may be critical for the disease activity and progression of RA resistant to MTX and LEF therapy. Therefore, it is critical to understand the mechanisms regulating these Th subsets and their cytokine production, specifically, the pro-inflammatory/pathogenic mechanisms of IL-22/Th22 within the target tissues of RA patients and animal models, since targeting Th22/IL-22 directly may aid design of efficient medicines for RA. In this regard, the impact of other synthetic or biological DMARDs, such as sulfasalazine and TNF-α blockers, on IL-22 production and frequencies of IL-22+Th cells in RA remains to be investigated.

In summary, this study not only corroborates findings in our previous study and those in animal model and fibroblast-like synoviocytes of RA that higher frequencies of IL-22^+^CD4^+^T cells and plasma IL-22 level served as bio-markers reflecting the disease activity and might critically promote RA development, but also reveals, for the first time, that targeting these immune components, particularly circulating Th22, Th17 cells and cytokines (IL-22 and IL-17) produced by these cells, may significantly benefit RA therapy. This study improves the mechanistic understanding of RA development and facilitates the design of novel RA therapies.

## Methods

### Patients and healthy control individuals (HC)

This study was designed according to the guidelines of the Declaration of Helsinki and all experimental protocols were approved by the Human Ethics Committee of Jilin University (Changchun, China). Written informed consent was obtained from all participants.

A total of 60 patients with new-onset RA admitted as in-patients to the First Hospital of Jilin University between March 2011 and October 2015 and 20 healthy gender-and age-matched controls from the out-patient clinic of the same hospital were enrolled. RA was diagnosed based on the revised RA classification criteria by the American College of Rheumatology^43^. Disease activity was assessed using the 28-joint disease activity score (DAS28). A score ≥2.6 qualifies the disease as active^44^. Patients with any of the following conditions were excluded: recent infection, a history of other autoimmune diseases such as myositis or systemic sclerosis, or a history of treatment with immunosuppressive agents or glucocorticoids within the past six months. Upon enrollment, peripheral blood samples were collected and all RA patients received MTX (10 mg/week, oral) together with LEF (20 mg daily, oral) for 12-weeks^45^. Following treatment, a second peripheral blood sample was collected from each patient, and treatment response was assessed using DAS28. Patients with a post-treatment DAS28 score <3.2 or a reduction of DAS28 > 1.2 were classified into the response group, while those with a DAS28 value of ≥3.2 and a reduction of DAS28 ≤ 1.2 were assigned to the non-response group.

### Data collection

The demographic and clinical data at baseline (before treatment), including age, sex, and current medications, were acquired from hospital records. Routine laboratory tests, including complete blood count, serum cyclic citrullinated peptide antibody (CCP), C-reactive protein (CRP), erythrocyte sedimentation rate (ESR), and rheumatoid factor (RF) were performed as previously described^15^.

### Isolation and activation of peripheral blood mononuclear cells (PBMCs)

Peripheral blood was collected from all participants after an overnight fast, and PBMCs were isolated as described previously^26^. PBMCs (10^6^ cells/ml) were stimulated in duplicate with phorbol 12-myristate 13-acetate (PMA, 1 μg/mL) and ionomycin (50 μg/mL; Sigma, St. Louis, MO, USA) in 10% human sera (AB type) in RPMI-1640 medium at 37 °C in a humidified incubator with 95% air and 5% carbon dioxide for 4 h, and cultured for another 2 h in the presence of brefeldin A (BFA, 0.5 μg/ml; Sigma). As the negative control, PBMCs not treated with PMA, BFA, and ionomycin were used.

### Flow cytometric analysis

Upon activation, PBMCs were detached, washed, and stained with peridinin chlorophyll (PerCP)-conjugated anti-CD4 antibody (Becton Dickinson, San Diego, CA, USA) at room temperature for 30 min, fixed with 4% paraformaldehyde and permeabilized using PBS containing 0.5% saponin and 10% fetal bovine serum (FBS) at room temperature for 30 min. After three washes, the cells were stained with fluorescein isothiocyanate (FITC)-conjugated anti-IFN-γ, Alexa-Fluor647-conjugated anti-IL-17 (Becton Dickinson), and phycoerythrin (PE)-conjugated anti-IL-22 (R&D Systems, Minneapolis, MN, USA). Flow cytometry was performed on a FACS Calibur (Becton Dickinson) and analyzed using the FlowJo software (TreeStar, San Carlos, CA, USA).

### Enzymen-linked immunosorbent assay (ELISA)

The plasma levels of IFN-γ, IL-17, and IL-22 were measured using ELISA kits (R&D Systems), according to the manufacturer’s instructions.

### Statistical analysis

Statistical analysis was performed using SPSS 21.0 (SPSS, Chicago, IL, USA). Quantitative data were presented as individual values or median (range) of each group. The differences between groups were analyzed using the Kruskal-Wallis ANOVA followed by Dunn-Bonferroni post hoc method or a Mann-Whitney U test when appropriate. Wilcoxon matched paired test was used for comparing pre- vs. post-treatment data. Correlation analysis was performed using Spearman’s rank correlation test. A two-sided P value of <0.05 was considered statistically significant.

## Additional Information

**How to cite this article**: Zhong, W. *et al*. IL-22-producing CD4+T cells in the treatment response of rheumatoid arthritis to combination therapy with methotrexate and leflunomide. *Sci. Rep.* **7**, 41143; doi: 10.1038/srep41143 (2017).

**Publisher's note:** Springer Nature remains neutral with regard to jurisdictional claims in published maps and institutional affiliations.

## Figures and Tables

**Table 1:** Demographic and clinical characteristics of RA patients and healthy controls (HC).

Parameters	RA patients	HC
	(n = 60)	(n = 20)
Age (years)	51 (35–79)	50 (30–75)
Gender ratio: female/male	41/19	12/8
RF (IU/mL)	72 (0.11–2922)*	11 (0.30–16.80)
CCP (U/mL)	397 (0.65–3250)*	18 (1.23–28.57)
ESR (mm/h)	39 (4–126)*	2 (0–5)
CRP (mg/L)	25 (0.61–226)*	6.9 (0–15)
DAS28	5.98 (3.29–9.33)	ND
WBC (10^9^/L)	6.72 (4.11–9.75)	5.76 (4.01–9.89)

Note: Data are presented as median (range) or number of cases. RA, rheumatoid arthritis; HC, healthy control; ND, non-detectable; RF, rheumatoid factor; CCP, cyclic citrullinated peptide antibody; DAS28, disease activity score of 28 joints; WBC, White blood cell counts; ESR, Erythrocyte sedimentation rate; CRP, C-reactive protein. Normal values: WBC: 3.50–9.50 × 10^9^/L, ESR: 0–15 mm/h, CRP: 0–3 mg/L, RF: 0–15 IU/mL; CCP: 0–25 U/mL. *P < 0.05 versus HC.

**Table 2:** Treatment with MTX and LEF improves the clinical profiles of RA patients.

Parameters	Response group (n = 40)		None-response group (n = 20)		HC (n = 20)
	Before treatment	After treatment	Before treatment	After treatment	
Age (years)	51 (36–79)	51 (36–79)	49 (35–78)	49 (35–78)	50 (30–75)
Sex ratio: female/male	27/13	27/13	14/6	14/6	12/8
RF (IU/mL)	350 (0.11–2922)^#^	72 (6–760)^*,△,#^	225 (16–2850)^#^	202 (13–1998)^#^	11 (0.30–16.80)
CCP (U/mL)	390 (0.65–2900)^#^	82 (0.50–890)^*,△,#^	385 (53.5–3250)^#^	336 (48–2500)^#^	18 (1.23–28.57)
ESR (mm/h)	38 (4–112)^#^	3 (0–5)*^,△^	40 (4–126)^#^	12 (4–68)^#^	2 (0–5)
CRP (mg/L)	27.22 (1.02–226)^#^	12.50 (0.87–79)*^,△^	17.23 (0.61–177)^#^	15.66 (2.06–29)^#^	6.9 (0–15)
DAS28	5.72 (3.29–8.26)	2.60 (1.82–3.05)*^,△^	6.92 (4.10–9.33)	6.23 (3.65–8.98)	ND
WBC (10^9^/L)	6.80 (4.13–9.75)	6.26 (4.36–8.89)	6.52 (4.11–9.74)	7.23 (4.07–9.89)	6.76 (4.01–9.89)

Note: Data are presented as median (range) or number of cases. MTX, methotrexate; LEF, leflunomide; RA, rheumatoid arthritis; HC, healthy control; ND, non-detectable; RF, rheumatoid factor; CCP, cyclic citrullinated peptide antibody; DAS28, disease activity score of 28 joints; WBC, White blood cell counts; ESR, Erythrocyte sedimentation rate; CRP, C-reactive protein. Normal values: WBC: 3.50–9.50 × 10^9^/L, ESR: 0–15 mm/h, CRP: 0–3 mg/L, RF: 0–15 IU/mL; CCP: 0–25 U/mL. *P < 0.05 versus before treatment in the same patient group. ^#^P < 0.05 versus HC. ^△^P < 0.05 versus non-response group at the same time point.
